# Reconsidering Carotid Artery Stenting for Asymptomatic Carotid Stenosis in the Era of Endovascular Evolution

**DOI:** 10.3390/biomedicines14030674

**Published:** 2026-03-16

**Authors:** Chloe DeYoung, Brandon Lucke-Wold

**Affiliations:** 1 College of Medicine, University of Florida, Gainesville, FL 32610, USA; 2 UF Health Department of Neurosurgery, Gainesville, FL 32608, USA

**Keywords:** carotid artery stenosis, endovascular neurosurgery, stenting

## Abstract

Management strategies in asymptomatic carotid artery stenosis are largely centered on intensive medical management, with carotid revascularization via endarterectomy or carotid artery stenting being reserved for select patients. This decision may be based on stenosis severity, perioperative risk, and patient preference. Current guidelines emphasize shared decision-making for patients with severe (>70%) stenosis, informed by prior trial data that does not demonstrate superiority of revascularization over independent medical therapy. Other studies more specifically recommend carotid endarterectomy over carotid artery stenting for asymptomatic patients with >60% stenosis. However, these studies are limited by poor statistical power. Recent findings in the CREST-2 trial have challenged this discussion of medical management as an independent primary course of action. The carotid artery stenting arm demonstrated significant long-term reduction in ipsilateral ischemic stroke compared to medical therapy alone. In this perspective, we argue that this new evidence supports a renewed role for carotid artery stenting in carefully selected patients with severe asymptomatic carotid artery stenting.

## 1. Introduction

Optimal treatment of asymptomatic carotid artery stenosis continues to be debated with options of intensive medical therapy or medical therapy with revascularization via carotid artery stenting or carotid endarterectomy as the main treatment options [1]. Earlier randomized trials evaluating this topic favor a conservative approach while the new CREST-2 trial shows statistical benefits with stenting [2,3,4,5] (Table 1). Plaque characteristics, patient life expectancy, risk factors for restenosis, and plan adherence may all introduce variables in consideration of revascularization [6,7,8,9,10,11]. Continued research in the area has prompted reconsideration of how asymptomatic carotid disease is defined, risk-stratified, managed, and where the future direction of research may lie.

## 2. Previous Trials Find No Benefit in Revascularization

A previous randomized trial, the SPACE-2 trial, sought to determine whether intensive medical management alone was comparable to medical therapy combined with revascularization either through endarterectomy or carotid artery stenting [2]. The trial data shows no statistical significance in stroke or death within the perioperative or the 5-year observation period during follow-up.

Importantly, SPACE-2 was underpowered, enrolling fewer than 16% of the intended study population over a decade of recruitment time [2]. With only 513 patients ultimately included, the trial was insufficiently powered to detect what we would expect to be a modest clinical difference with revascularization as seen in other studies. Although this difference would be modest, it can still be clinically significant when pooling yearly risk over the rest of a life expectancy. In this study, while both medical therapy and revascularization performed well in comparison to historical outcomes, the study design limited the ability to discern incremental benefit from placing a carotid stent [2].

Similarly, the ECST-2 trial compared medical therapy alone with medical therapy plus revascularization in patients with carotid stenosis greater than 50%, without restricting enrollment to asymptomatic patients [4]. At two years, no significant difference in clinical outcomes was observed, including among patients with severe (>70%) stenosis. However, imaging endpoints did reveal a higher incidence of new ischemic brain lesions on MRI in the medical therapy group than with the revascularization group. Although this data was not powered for definitive conclusions, these findings suggest potential subclinical benefits that may not be captured statistically by short-term clinical endpoints alone.

Each of these trials has a limitation of relatively short follow-up times [2,4,5]. When discussing annual risks that will continue to accrue over a lifetime, short-term follow-up may not elucidate long-term risks of a lack of intervention. Asymptomatic carotid artery stenosis has a modest annual stroke risk, therefore in the short term we would expect that medical management versus intervention-based therapy may not realistically portray a large difference statistically and that the superiority of one intervention may be better elucidated in long-term intervals. This distinction in follow-up duration may be specifically pertinent when considering patients with a substantial life expectancy with an expected larger risk accrual.

Collectively, these trials shape a conservative approach to carotid stenting, with data that supports proper medical management as comparable to revascularization. However, there is not a demonstration of inferiority of revascularization, and methodological and temporal limitations may have left the clinical question incompletely resolved [2,4].

## 3. CREST-2 as a Turning Point

The CREST-2 trial, published in November 2025, re-examined the use of revascularization in asymptomatic severe carotid artery stenosis via parallel trials with carotid endarterectomy or carotid artery stenting alongside medical management as opposed to intensive medical management alone [5]. Perioperative stroke or death were evaluated as well as four-year ipsilateral ischemic stroke outcomes in a total of 1245 patients across 155 centers.

While the carotid endarterectomy arm did not demonstrate a statistically significant superiority to independent medical management, the carotid artery stenting arm of the study did have data demonstrating a meaningful risk reduction [5]. Among patients undergoing carotid artery stenting, the four-year incidence of stroke or death was significantly lower than in those managed with medical therapy alone, corresponding to an absolute risk reduction of 3.2% and a number needed to treat of 31 [5].

Although there was higher perioperative risk seen which was modest in the stenting group, the long-term outcomes favor revascularization [5]. Annualized rates of ipsilateral ischemic stroke risk were 1.7% in the medical management group which dropped to 0.4% in patients with revascularization [5]. This reduction in yearly risk offers clinically meaningful stroke risk reduction in appropriate patients. However, when theorizing long-term outcomes past the 4-year evaluation point, it is also important to keep in mind the risk of restenosis at the location of the stent. Data from the CREST trial was used to look at restenosis, showing 6.0% of stenting patients and 6.3% of carotid endarterectomy patients had restenosis or occlusion after two years [6]. Of these patients, female sex, diabetes, and dyslipidemia were predictors of restenosis in CAS, supporting continued use of best medical therapy, reduction in comorbidities, and adequate follow-up postoperatively [6]. The benefit of CAS may be of greater magnitude in patients with longer life expectancies as these annual risks accrue over the following years into a substantial lifetime stroke risk reduction. In contrast, patients with higher operative risk, lower life expectancies, or comorbidities relating to restenosis may not derive the same lifetime benefit from revascularization. Again, highlighting the importance of appropriate patient selection.

Other factors worth considering in terms of endovascular intervention and patient education would be patient anatomy. Favorable aortic arch anatomy, absence of extensive vessel tortuosity, and lesion characteristics may lend themselves better to an endovascular approach [7,8]. Plaque morphology, specifically echo-lucent plaques, ulceration, or presence of intraplaque hemorrhage, carry an increased risk of embolism even in currently asymptomatic patients [8]. Transcranial Doppler (TCD) and near-infrared spectroscopy (NIRS) may also be used as a non-invasive measure to evaluate cerebral perfusion and vasomotor activity in patients with carotid artery stenosis during hemodynamic challenge [9,10]. Further imaging may be necessary to determine plaque morphology and circulatory status to determine a more accurate delineation of individual risk to the patient aside from their categorization as asymptomatic. These patients should be counseled appropriately and offered appropriate intervention.

Finally, institutional expertise plays a critical role [12]. Outcomes of carotid artery stenting are closely linked to operator experience and procedural volume. This was specifically noted in the CAPTURE 2 study with an inverse relationship noted between operator volume and adverse events [12]. Using only operators with proven success in these stenting trials introduces poor external validity for similar low perioperative risks in facilities with lower experience. However, these findings support increased offerings of these procedures at experienced institutions with demonstrated low periprocedural complication rates as well as continued training of upcoming physicians in these methods.

## 4. The Role of Medical Management

It is important to acknowledge that intensive medical management alone does provide a relatively low annual stroke risk in these patients with severe asymptomatic carotid artery stenosis, when the medical management is adhered to and optimized [2,3,4,5,13,14]. Patients may prefer this option to avoid procedural risk and that should be offered to them. Although there were some perioperative strokes in the carotid stenting arm of CREST-2, the overall incidence was low, and they were largely non-disabling [5]. This information should also be shared with patients to help contextualize what that risk may look like, which may mitigate concerns of operation.

In addition to overall stroke incidence, the functional impact of these events warrants consideration when weighing revascularization against medical therapy alone. Not all strokes carry equivalent clinical consequences, and even small reductions in ischemic stroke risk may translate into meaningful preservation of neurologic function, independence, and quality of life.

In CREST-2, the majority of perioperative strokes associated with carotid artery stenting were non-disabling with a theoretically diminished lifetime risk, whereas ischemic strokes occurring during longitudinal follow-up in medically managed patients carry the potential for cumulative neurologic injury over time [5]. While the trial was not powered to detect differences in functional outcomes, prevention of even minor strokes may have exaggerated importance in younger or professionally active patients. These considerations emphasize that stroke prevention strategies should be evaluated not only by event rates, but also by their long-term functional implications.

Another commonly cited limitation of CREST-2 as well as SPACE-2 is their reliance on experienced operators with documentation of low perioperative complication rates [2,5]. This can potentially limit generalizability to broader clinical practice. However, procedural outcomes are inherently linked to provider experience, and increased adoption of carotid artery stenting would likely be accompanied by further refinement of techniques alongside improved outcomes.

## 5. Limits of Generalizability of Intensive Medical Management

When considering intensive medical management implemented in controlled trials, it is essential to consider generalizability. Participants in CREST-2 received structured support via health coaching and close monitoring with optimization of many medical factors [5]. These resources may not be readily available for the general public or in routine practice. Trial participants may also represent a more highly motivated subset of patients that will follow recommendations with a greater level of adherence.

In the real world, suboptimal adherence is more likely with intensive medical therapy, and this could attenuate the protective effects of intensive medical management. It is imperative to keep that in mind when extrapolating these results to clinical decision-making.

## 6. Extra Clues: Advanced Imaging and Risk Stratification

Advanced imaging techniques can be useful to further elucidate the risk level of an individual patient’s situation regarding the morphology of their plaque in the presence or absence of symptoms [11]. MRI can be used to further delineate plaque morphology in identifying intraplaque hemorrhage, ulceration, lipid-rich necrotic core, and inflammation [8,11]. CT can also detect ulceration and calcification with limited lipid-rich necrotic core and intraplaque hemorrhage detection. These radiologic findings challenge binary classification of carotid stenosis as either symptomatic or asymptomatic by providing more tools for physicians to use in assessing risk of adverse events for their patients.

These biomarkers should also be used aside from symptoms and degree of stenosis to work with your patient for the best clinical outcomes [8,11]. Incorporating this into clinical decision-making can refine patient selection and align interventions with individualized risk profiles. It is important to keep in mind that asymptomatic does not equal low risk in every case and that readily available imaging data can be used in adjunct to other endovascular-approach decision factors.

## 7. Moving Forward: Redefining the Role of Carotid Artery Stenting

Management of asymptomatic severe carotid artery stenosis continues to evolve alongside advancements in both medical therapy as well as endovascular technique and skill. Prior trials favored a more conservative approach due to lack of superiority of revascularization demonstrated in trials [2,4]. The CREST-2 trial represents a methodologically robust reassessment of revascularization with a higher statistical power suggesting carotid artery stenting provides a meaningful reduction in long-term stroke risk and can be useful in select patients [5].

These findings should drive further research into whether long-term data supports a cumulative decreased risk of stroke which may prompt alteration in guideline recommendations, particularly for this subset of patients with asymptomatic severe stenosis and sufficient life expectancy where there will be substantial cumulative long-term ischemic stroke risk reduction [5]. Moving the observation period longer than the timeline included in the current trials may help strengthen these arguments. Those with elevated operative risk factors or significant risks for restenosis may not be as great of candidates [6].

Additionally, the term “asymptomatic” itself may oversimplify a biologically heterogeneous disease. The absence of clinical symptoms does not necessarily relate to an absence of risk. Adjunct radiologic findings suggest that certain patients categorized as asymptomatic still carry lesions with substantial embolic risk or have poor cerebral perfusion under hemodynamic distress [8,9,10]. Reframing asymptomatic carotid stenosis as a spectrum of risk with multiple contributions that may characterize it, rather than a binary designation, may more accurately reflect our current understanding of this disease process and support more individualized treatment strategies. Future research can focus on refining this patient selection criteria as well as assessing durability of this reduced risk as these stented patients continue to be monitored.

Beyond individual patient outcomes, the management of asymptomatic carotid artery stenosis carries broader health system implications. Ischemic stroke remains a leading cause of long-term disability, with substantial downstream costs related to rehabilitation, loss of productivity, and chronic care needs [15]. Preventing a proportion of these strokes in appropriately selected patients may reduce overall healthcare costs and have societal benefits over time. While carotid artery stenting incurs upfront procedural costs, these must be contextualized against the economic and personal burden of long-term stroke-related morbidity. Future cost-effectiveness analyses may further clarify the value of revascularization in selected asymptomatic populations [15].

As endovascular technology continues to advance, carotid artery stenting should be viewed not as a competing alternative to medical therapy, but as a complementary option within an individualized, evidence-informed treatment framework focused on optimal clinical outcomes.

## Figures and Tables

**Table 1:** Summary of severe asymptomatic artery stenosis trial data. BMT (best medical therapy); CEA (carotid endarterectomy); CAS (carotid artery stenting).

Trial	ECST-2 [3]	SPACE-2 [2]	CREST-2 [5]
Sample Size	429	513 (goal of 3550)	1245 in CAS trial 1240 in CEA trial
Treatment Arms	BMT vs. BMT with revascularization (CEA or CAS)	BMT alone vs. CEA + BMT vs. CAS + BMT	1–BMT + CAS vs. BMT alone 2–BMT + CEA vs. BMT alone
Primary Outcome	Periprocedural death/fatal stroke/fatal MI Non-fatal stroke Non-fatal MI New silent infarction	Any stroke/death within 30 days OR ipsilateral ischemic stroke within 5 years	Periprocedural stroke/death OR post-procedural ipsilateral stroke within 4 years
Follow-Up Duration	2 years (interim analysis)	5 years	4 years
Key Results	No benefit of revascularization over BMT alone.	No superiority of re-vascularization over BMT alone.	Stenting trial shows statistical benefit. (*p* = 0.22, NNT = 31) No benefit of CEA over BMT alone.

## Abbreviations

The following abbreviations are used in this manuscript:

BMT	Best Medical Therapy
CEA	Carotid Endarterectomy
CAS	Carotid Artery Stenting
TCD	Transcranial Doppler
NIRS	Near-Infrared Spectroscopy
